# The Diagnostic Accuracy of HE4 in Lung Cancer: A Meta-Analysis

**DOI:** 10.1155/2015/352670

**Published:** 2015-03-19

**Authors:** Daye Cheng, Ying Sun, Hu He

**Affiliations:** Department of Transfusion, First Hospital of China Medical University, Shenyang 110001, China

## Abstract

The diagnostic value of serum HE4 in patients with lung cancer remains controversial. Thus, we performed a systematic review and meta-analysis to assess the diagnostic accuracy of serum HE4 for lung cancer. We conducted a comprehensive literature search in PubMed, EMBASE, Chinese National Knowledge Infrastructure (CNKI), and WANFANG databases between Jan. 1966 and Nov. 2014. The diagnostic sensitivity (SEN), specificity (SPE), positive likelihood ratio (PLR), negative likelihood ratio (NLR), diagnostic odds ratio (DOR), and summary receiver operating characteristic curve (SROC) were pooled by Meta-DiSc 1.4 software. A total of seven articles including 715 cases and 549 controls were included for analysis. The summary estimates for serum HE4 in the diagnosis of lung cancer in these studies were pooled SEN 0.72 (95% CI: 0.68–0.75), SPE 0.85 (95% CI: 0.81–0.88), PLR 4.68 (95% CI: 3.23–6.78), NLR 0.31 (95% CI: 0.24–0.39), and DOR 17.14 (95% CI: 9.72–30.20), and the area under the curve (AUC) was 0.8557. This meta-analysis indicated that serum HE4 is a potential tool in the diagnosis of lung cancer. In addition, considering the high heterogeneity and potential publication bias, further studies with rigorous design and large sample size are needed in the future.

## 1. Introduction

Lung cancer is a leading cause of cancer morbidity and mortality worldwide. According to statistics from the National Office of Tumor Cure and Prevention, about 600,000 people die of lung cancer each year in China, accounting for approximately 20% of all cancer deaths [1, 2]. Despite advances in the diagnosis and treatment of lung cancer, the prognosis of lung cancer is still poor, with around 16% surviving 5 years after diagnosis [3]. Considering that the 5-year survival of stage I in lung cancer is as high as 83% [4], early diagnosis is essential to reduce mortality of this fatal disease. To date, circulating tumor markers for lung cancer have become a major focus. Lines of evidence demonstrated that serum carcinoembryonic antigen (CEA), neuron specific enolase (NSE), cytokeratin fragment (CYFRA21-1), tissue polypeptide specific antigen (TPS), and progastrin-releasing peptide (ProGRP) were believed as potential markers to diagnosis of lung cancer [5–10]. However, due to low sensitivity and specificity, the clinical values of them are limited. Therefore, early and accurate diagnostic tool for lung cancer is especially important for lung cancer management.

Human epididymis protein 4 (HE4), encoded by WAP 4-disulfide core domain 2 (*WFDC2*) [11], was first identified in the epithelium of the distal epididymis and originally predicted to be a protease inhibitor involved in sperm maturation [12]. To date, overexpression of HE4 has been demonstrated in a range of malignant neoplasms, especially of gynecological and pulmonary origin [13]. In 2011, Yamashita et al. reported that serum HE4 had a significantly high diagnostic sensitivity and specificity for lung cancer [14]. Although the extensive analyses have been carried out, owing to the limitation of relatively small patient population and heterogeneous patient type, the application of HE4 in early diagnosis of lung cancer still needs to be validated and thoroughly investigated in larger studies.

To fully understand the diagnostic performance of serum HE4 for lung cancer, we performed a systematic review and meta-analysis to evaluate the role of HE4 in the diagnosis of lung cancer.

## 2. Materials and Methods

### 2.1. Literature Search

A systematic search was carried out to identify studies assessing the diagnostic value of HE4 for human lung cancer. The PubMed, EMBASE, Chinese National Knowledge Infrastructure (CNKI), and WANFANG databases were searched for articles that were published between Jan. 1966 and Nov. 2014. The key words were as follows: “lung tumor” OR “lung cancer” OR “lung carcinoma” AND “Human epididymis protein 4” OR “HE4” OR “WFDC2” AND “blood” OR “serum” OR “circulating” AND “diagnosis” OR “sensitivity and specificity” OR “ROC curve.”

### 2.2. Inclusion and Exclusion Criteria

Eligible studies included in this meta-analysis have to fulfill the following criteria: (1) studies regarding the diagnostic potential of circulating HE4 for lung cancer; (2) studies with a gold reference standard for lung cancer diagnosis; (3) sensitivity and specificity of HE4 being reported to provide sufficient information to construct two × two contingency tables. Exclusion criteria were (1) studies with ambiguous diagnostic criteria; (2) studies with duplicate data reported in other studies; and (3) studies that were published as reviews, letters, case reports, editorials, or comments.

### 2.3. Data Extraction and Quality Assessment

Two investigators (Cheng and Sun) independently extracted the following information from the eligible studies: author, year of publication, country of origin, sample size, assay methods, cut-off values, and diagnostic performance (sensitivity, specificity, TP, FP, FN, and TN). The disagreements on eligibility of studies were resolved by full-text review and discussion.

The Quality Assessment of Diagnostic Accuracy Studies (QUADAS) was used to assess each study for the quality of the information reported [21]. QUADAS is a quality assessment tool specifically developed for systematic reviews of diagnostic accuracy studies to assess bias in the study, including 14 questions (each of which is scored as yes, no, or unclear).

### 2.4. Statistical Analysis

The STATA software, version 12.0 (Stata Corporation, College Station, TX) and Meta-DiSc 1.4 (XI Cochrane Colloquium, Barcelona, Spain) were used to perform all data analysis. The bivariate meta-analysis model was employed to summarize the sensitivity (SEN), specificity (SPE), positive likelihood ratio (PLR), negative likelihood ratio (NLR), and diagnostic odds ratio (DOR). The SEN and SPE of each included study were used to plot the summary receiver operator characteristic (SROC) curve and calculate the area under the SROC curve (AUC). The between-study heterogeneity was evaluated by *Q* test and *I* ^2^ statistics. A *P* < 0.10 for *Q* test or *I* ^2^ value >50% indicates substantial heterogeneity, and the random effects model was applied; otherwise, fixed-effects model was adopted. Additionally, the Spearman approach was applied to verify whether the heterogeneity in meta-analysis could be explained by threshold effect [22]. The presence of publication bias was assessed by using the funnel plot followed by Egger's test analysis [23]. A *P* value less than 0.05 was considered statistically significant.

## 3. Results

### 3.1. Study Characteristics

**Figure 1:**
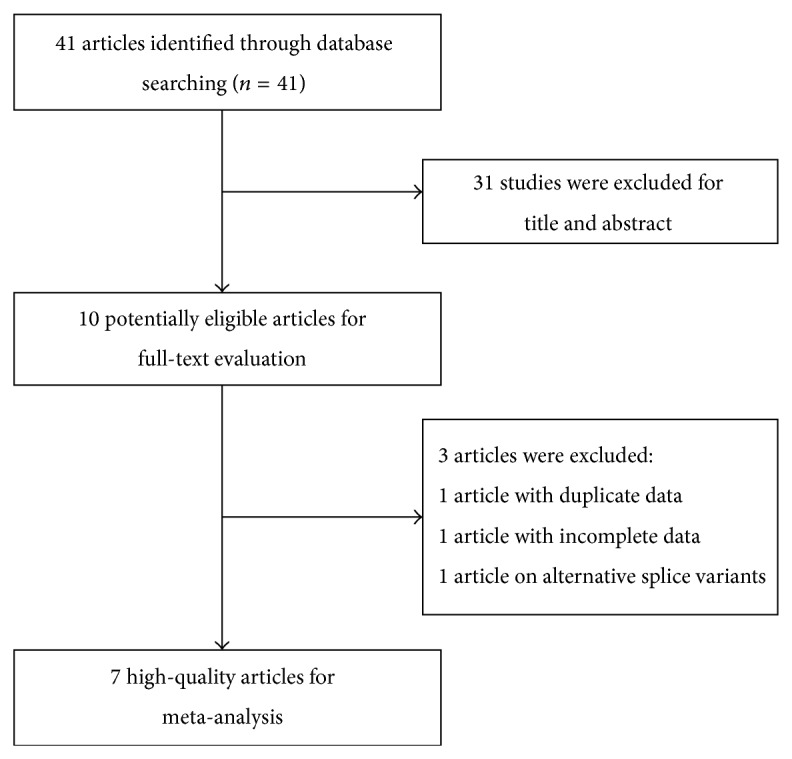
Flow chart describing systematic literature search and study selection process.

The detailed flow diagram of literature retrieval was presented in Figure 1. A total of 41 potentially relevant articles were retrieved after initial databases search. Of 41 searched articles, we excluded 31 articles that were not relevant to our study on the basis of title and abstract. After reviewing the full-text, only seven articles with 715 cases and 549 controls were included for systematic review and meta-analysis [14–20]. The characteristics of the included studies were shown in Table 1. The sample size ranged from 78 to 290. Six studies were performed in Asian [14–16, 18–20] and one in European [17].

### 3.2. Quality Assessment

Quality assessment based on QUADAS guidelines was conducted on all 7 studies included for systematic review. The QUADAS scores of studies were from 11 to 13 which satisfy the majority of the standards.

### 3.3. Data Analysis

**Figure 2:**
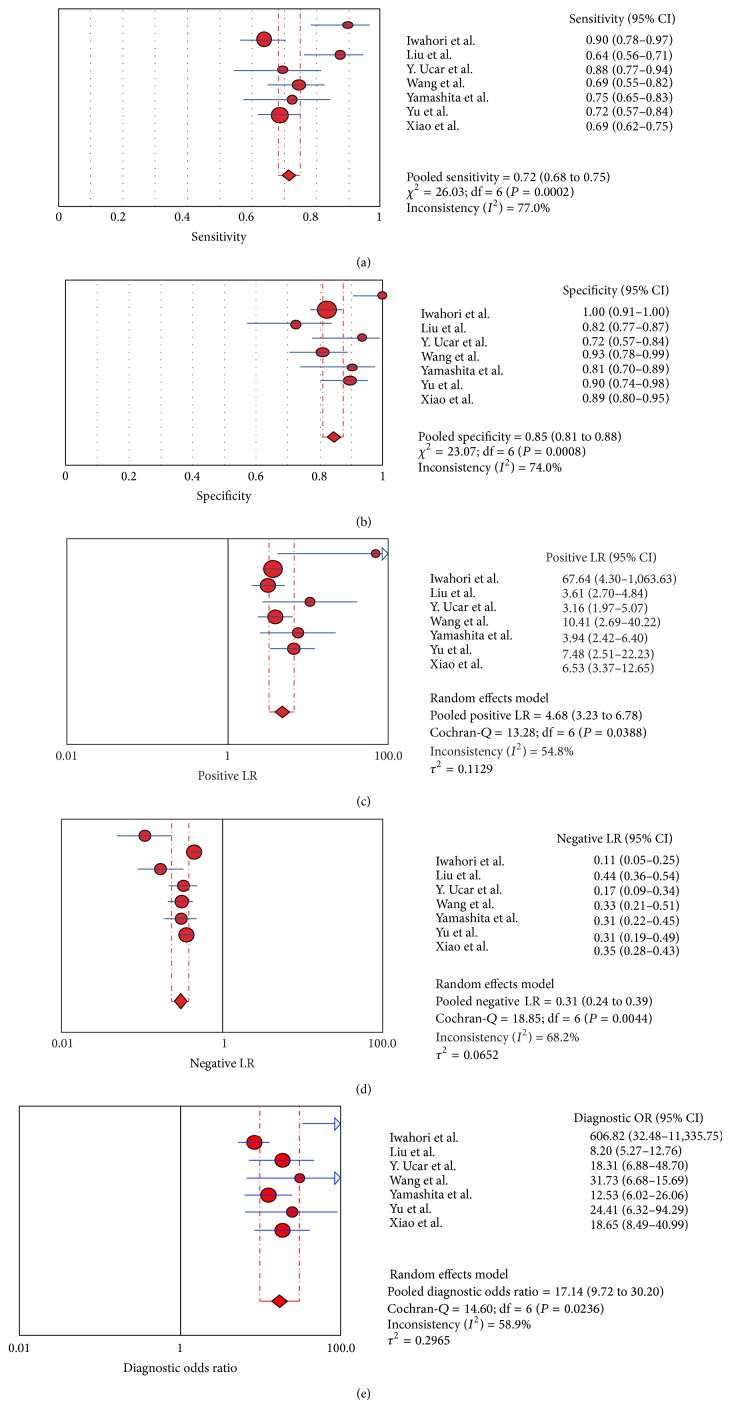
Forest plot of the sensitivity, specificity, positive likelihood ratio (PLR), negative likelihood ratio (NLR), and diagnostic odds ratio (DOR) in lung cancer diagnosis. (a) Sensitivity; (b) specificity; (c) PLR; (d) NLR; and (e) DOR.

The forest plot of SEN, SPE, PLR, NLR, and DOR for HE4 in the diagnosis of lung cancer was shown in Figure 2. By heterogeneity analysis, *I* ^2^ of SEN, SPE, PLR, NLR, and DOR was 77% (*P* < 0.001), 74.0% (*P* < 0.001), 54.8% (*P* = 0.039), 68.2% (*P* = 0.004), and 58.9% (*P* = 0.024), respectively, implicating significant heterogeneity of the studies. Therefore, the random effects model was selected in this study for further analysis. To verify whether the heterogeneity could be explained by a threshold effect, the Spearman approach was applied. Spearman correlation coefficient of these 7 articles was −0.071 (*P* = 0.879), suggesting that there was no significant threshold effect.

**Figure 3:**
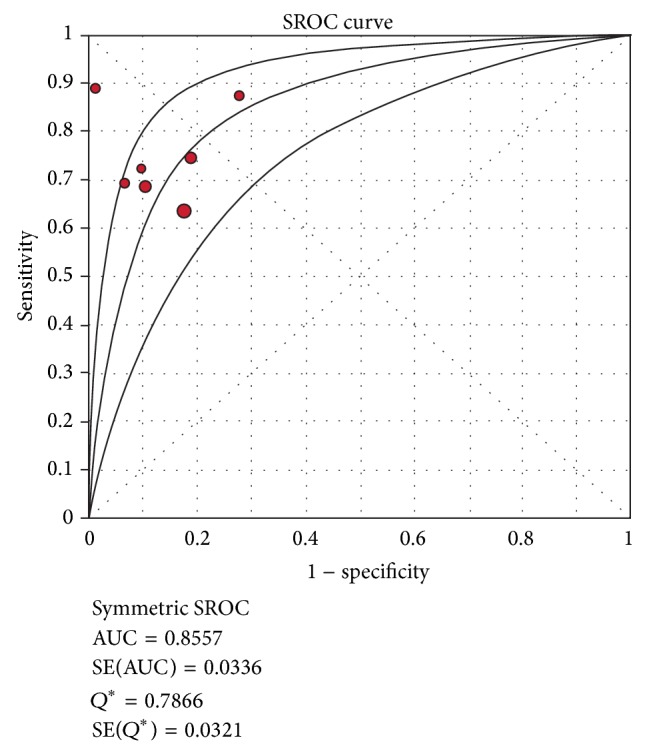
Summary receiver operating characteristic curves (SROC) of HE4 in the diagnosis of lung cancer.

The pooled SEN of HE4 for the diagnosis of lung cancer was 0.72 (95% confidence interval (CI), 0.68–0.75) (Figure 2(a)) and the pooled SPE was 0.85 (95% CI, 0.81–0.88) (Figure 2(b)), respectively. The PLR was 4.68 (95% CI, 3.23–6.78) (Figure 2(c)), the NLR was 0.31 (95% CI, 0.24–0.39) (Figure 2(d)), and the DOR was 17.14 (95% CI, 9.72–30.20) (Figure 2(e)), respectively. These results demonstrated that HE4 was an effective diagnostic marker for lung cancer. SROC results showed that AUC of HE4 was 0.8557, indicating that HE4 may be able to differentiate lung cancer patients from non-lung cancer patients with a relatively high accuracy (Figure 3).

### 3.4. Publication Bias

**Figure 4:**
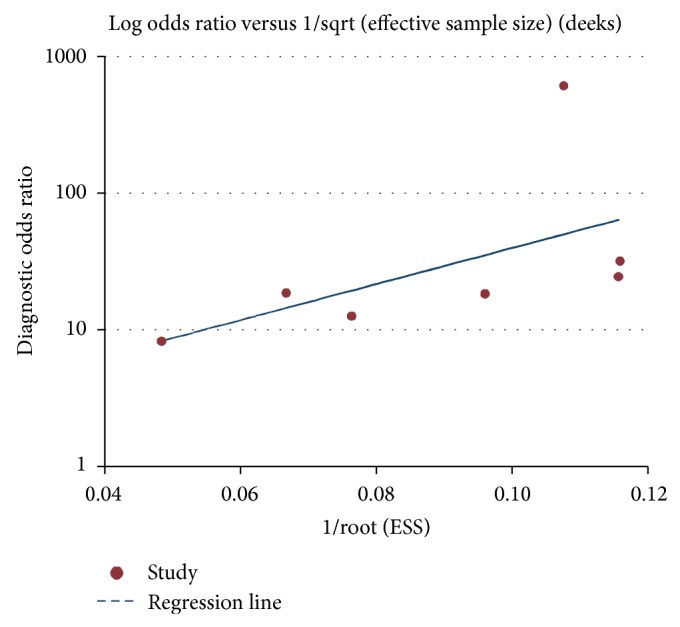
Funnel plot for the evaluation of potential publication bias in diagnosis of HE4 for lung cancer.

To investigate the publication bias, we performed the funnel plot and Egger's test. The shape of funnel plot did not reveal any evidence of obvious asymmetry (Figure 4). The result of Egger's test was 0.090, suggesting no publication bias among the included studies for HE4 in the diagnosis of lung cancer.

## 4. Discussion

The diagnosis of lung cancer remains a clinical challenge. Growing evidence suggests that serum HE4 has emerged as a promising biomarker for lung cancer diagnosis, but with considerable varying results. Meta-analysis is a powerful tool for summarizing the results from different studies by producing a single estimate of the major effect with enhanced precision and reducing random error [24]. This meta-analysis involving 715 cases and 549 controls from 7 studies provides suggestive evidence that serum HE4 is a potential marker for lung cancer diagnosis.

*HE4 *encodes for a highly conserved WAP domain-containing protein, which is suggestive of putative serine protease inhibitor activity [25]. The exact function of HE4 is poorly understood, but its status as likely extracellular protease inhibitors suggests that they may be involved in the regulation of extracellular matrix, cell migration, and cell invasion. Accumulating evidence has demonstrated that HE4 is frequently overexpressed in ovarian cancer, but some expression has also been found in lung [14], endometrial [26], and breast cancer [27] and, less often, gastric [28], pancreas [28], and transitional cell carcinomas [29]. Moreover, Drapkin et al. suggested that HE4 is a secreted glycoprotein and is present in the circulation, which may play an important role in the biology of cancer [30]. Until now, HE4 has gained widespread use as a soluble tumor marker in the diagnosis and follow-up of patients with ovarian cancer [31, 32]. Recently, many medical researchers investigated the potential of HE4 as a serum biomarker for diagnosis of lung cancer, but the results remain controversial. The present meta-analysis indicated that the sensitivity and specificity of HE4 were 0.72 and 0.85, respectively. This means that 72% of the lung cancer patients had high HE4 levels, and 85% of non-lung cancer patients have low HE4 levels. Compared with the conventional serum biomarkers, such as CEA, CYFRA21-1, and NSE [33], HE4 demonstrates higher sensitivity and specificity according to our recent study. DOR combines the strengths of sensitivity and specificity as prevalence in dependent indicators and is useful from the statistical point of view in the assessment of the overall test accuracy in meta-analysis [34]. The value of DOR ranges from 0 to infinity, with higher values indicating better discriminatory test performance [35]. In the present analysis, the mean DOR was 17.14, indicating that the odds for positivity among subjects with lung cancer were 17.14 times higher than the odds for positivity among non-lung cancer subjects. The SROC curve has been recommended to be a global indicator for assessing the diagnostic performance of index test, and it shows the trade-off between sensitivity and specificity [36]. The present meta-analysis found that the AUC was 0.8557 for HE4, also indicating that HE4 was an effective biomarker for lung cancer diagnosis.

Heterogeneity is a potential problem when interpreting the results of all meta-analysis [37]. In the present study, we found great heterogeneity among the included studies, and the result of Spearman approach showed that heterogeneity could not be explained by a threshold effect. We speculated that the heterogeneity was attributed to the ethnicity, etiology, assay methods, different geographical locations, and different stages of lung cancer patients. Due to the limited number of eligible studies, we could not further detect the source of heterogeneity. However, these hypotheses need to be investigated in the future study.

This meta-analysis also had additional limitations. First, we only included 7 studies that have a limited number of cases, which might have impaired the statistical power of analysis. Therefore, results of our meta-analysis need to be confirmed by well-designed studies with larger sample sizes. Second, the significant statistical heterogeneity could be found among our included studies, which might lead to the existence of bias factors. Third, we did not calculate the diagnostic accuracy for the different stages for lung cancer because sufficient raw data was not provided. Moreover, primary data were unavailable for evaluation of HE4 values as a function of tumor type, histology, age, or degree. Fourth, only the articles published in English and Chinese were included in this meta-analysis. This would give rise to inevitable bias. Fifth, only serum HE4 levels were evaluated in the meta-analysis which may be insufficient to confirm our viewpoints; hence, additional research on other tumor markers in the same samples could provide more valuable information.

## 5. Conclusion

In conclusion, the current evidence suggests that serum HE4 is a useful biomarker for lung cancer diagnosis. However, our conclusion needs to be confirmed by more well-designed research studies in order to confirm the significance of serum HE4 as a diagnostic indicator of lung cancer.

## Figures and Tables

**Table 1:** Summary of included studies.

Author	Year	Country	Case/controls	Assay method	Cut-off	TP	FP	FN	TN	QUADAS
Iwahori et al. [15]	2012	Japan	49/37	ELISA	6.56 ng/mL	44	0	5	37	13
Liu et al. [16]	2013	China	190/244	ELISA	82.61 pmol/L	121	43	69	201	12
Ucar et al. [17]	2014	Turkey	64/38	ELISA	67.5 pmol/L	56	13	8	34	12
Wang et al. [18]	2014	China	49/30	ELISA	84.19 pmol/L	34	2	15	28	11
Yamashita et al. [14]	2011	Japan	102/74	ELISA	50.3 pmol/L	76	14	26	60	12
Yu et al. [19]	2014	China	47/31	ELISA	149.145 pmol/L	34	3	13	28	11
Xiao et al. [20]	2014	China	214/76	ELISA	NR	147	8	67	68	12

TP: true positive; FP: false positive; TN: true negative; FN: false negative; NR: not reported; ELISA: enzyme-linked immunosorbent assay.

## References

[1] She J., Yang P., Hong Q., Bai C. (2013). Lung cancer in China: challenges and interventions. *Chest*.

[2] Lan Q., Chapman R. S., Schreinemachers D. M., Tian L., He X. (2002). Household stove improvement and risk of lung cancer in Xuanwei, China. *Journal of the National Cancer Institute*.

[3] Jemal A., Siegel R., Xu J., Ward E. (2010). Cancer statistics, 2010. *CA Cancer Journal for Clinicians*.

[4] Jemal A., Siegel R., Ward E. (2008). Cancer statistics, 2008. *CA—Cancer Journal for Clinicians*.

[5] Grunnet M., Sorensen J. B. (2012). Carcinoembryonic antigen (CEA) as tumor marker in lung cancer. *Lung Cancer*.

[6] Tufman A., Huber R. M. (2009). Biological markers in lung cancer: a clinician's perspective. *Cancer Biomarkers*.

[7] Kulpa J., Wójcik E., Radkowski A., Kolodziejski L., Stasik Z. (2000). CYFRA 21-1, TPA-M, TPS, SCC-Ag and CEA in patients with squamous cell lung cancer and in chemical industry workers as a reference group. *Anticancer Research*.

[8] Lamy P.-J., Grenier J., Kramar A., Pujol J.-L. (2000). Pro-gastrin-releasing peptide, neuron specific enolase and chromogranin A as serum markers of small cell lung cancer. *Lung Cancer*.

[9] Nisman B., Biran H., Ramu N., Heching N., Barak V., Peretz T. (2009). The diagnostic and prognostic value of ProGRP in lung cancer. *Anticancer Research*.

[10] Wójcik E., Kulpa J. K., Sas-Korczyńska B., Korzeniowski S., Jakubowicz J. (2008). ProGRP and NSE in therapy monitoring in patients with small cell lung cancer. *Anticancer Research*.

[11] Clauss A., Lilja H., Lundwall Å. (2002). A locus on human chromosome 20 contains several genes expressing protease inhibitor domains with homology to whey acidic protein. *Biochemical Journal*.

[12] Kirchhoff C. (1998). Molecular characterization of epididymal proteins. *Reviews of Reproduction*.

[13] Escudero J. M., Auge J. M., Filella X., Torne A., Pahisa J., Molina R. (2011). Comparison of serum human epididymis protein 4 with cancer antigen 125 as a tumor marker in patients with malignant and nonmalignant diseases. *Clinical Chemistry*.

[14] Yamashita S.-I., Tokuishi K., Hashimoto T. (2011). Prognostic significance of HE4 expression in pulmonary adenocarcinoma. *Tumor Biology*.

[15] Iwahori K., Suzuki H., Kishi Y. (2012). Serum HE4 as a diagnostic and prognostic marker for lung cancer. *Tumour Biology*.

[16] Liu W., Yang J., Chi P. D. (2013). Evaluating the clinical significance of serum HE4 levels in lung cancer and pulmonary tuberculosis. *International Journal of Tuberculosis and Lung Disease*.

[17] Ucar E. Y., Ozkaya A. L., Araz O. (2014). Serum and bronchial aspiration fluid HE-4 levels in lung cancer. *Tumor Biology*.

[18] Wang X., Fan Y., Wang J., Wang H., Liu W. (2014). Evaluating the expression and diagnostic value of human epididymis protein 4 (HE4) in small cell lung cancer. *Tumor Biology*.

[19] Yu F., Wang Q., Zhong D. (2014). The significance of serum HE4 levels in the diagnosis of lung cancer. *Tianjin Medical Journal*.

[20] Xiao Y., Hu H., Wang R. (2014). Investigation on serum HE4 levels in the diagnosis of lung cancer. *Laboratory Medicine*.

[21] Whiting P., Rutjes A. W. S., Reitsma J. B., Bossuyt P. M. M., Kleijnen J. (2003). The development of QUADAS: a tool for the quality assessment of studies of diagnostic accuracy included in systematic reviews. *BMC Medical Research Methodology*.

[22] Moses L. E., Shapiro D., Littenberg B. (1993). Combining independent studies of a diagnostic test into a summary ROC curve: data-analytic approaches and some additional considerations. *Statistics in Medicine*.

[23] Egger M., Smith G. D., Schneider M., Minder C. (1997). Bias in meta-analysis detected by a simple, graphical test. *British Medical Journal*.

[24] Yin D., Jiang Y., Wang N. (2014). The diagnostic value of serum hybrid capture 2 (CH2) HPV DNA in cervical cancer: a systematic review and meta-analysis. *Tumor Biology*.

[25] Clauss A., Lilja H., Lundwall Å. (2005). The evolution of a genetic locus encoding small serine proteinase inhibitors. *Biochemical and Biophysical Research Communications*.

[26] Jiang S.-W., Chen H., Dowdy S. (2013). HE4 transcription- and splice variants-specific expression in endometrial cancer and correlation with patient survival. *International Journal of Molecular Sciences*.

[27] Kamei M., Yamashita S.-I., Tokuishi K. (2010). HE4 expression can be associated with lymph node metastases and disease-free survival in breast cancer. *Anticancer Research*.

[28] O'Neal R. L., Nam K. T., Lafleur B. J. (2013). Human epididymis protein 4 is up-regulated in gastric and pancreatic adenocarcinomas. *Human Pathology*.

[29] Xi Z., LinLin M., Ye T. (2009). Human epididymis protein 4 is a biomarker for transitional cell carcinoma in the urinary system. *Journal of Clinical Laboratory Analysis*.

[30] Drapkin R., von Horsten H. H., Lin Y. (2005). Human epididymis protein 4 (HE4) is a secreted glycoprotein that is overexpressed by serous and endometrioid ovarian carcinomas. *Cancer Research*.

[31] Macedo A. C., da Rosa M. I., Lumertz S., Medeiros L. R. (2014). Accuracy of serum human epididymis protein 4 in ovarian cancer diagnosis: a systematic review and meta-analysis. *International Journal of Gynecological Cancer*.

[32] Piovano E., Attamante L., Macchi C. (2014). The role of HE4 in ovarian cancer follow-up: a review. *International Journal of Gynecological Cancer*.

[33] Chu X.-Y., Hou X.-B., Song W.-A., Xue Z.-Q., Wang B., Zhang L.-B. (2011). Diagnostic values of SCC, CEA, Cyfra21-1 and NSE for lung cancer in patients with suspicious pulmonary masses: a single center analysis. *Cancer Biology & Therapy*.

[34] De Sousa M. R., Ribeiro A. L. P. (2009). Systematic review and meta-analysis of diagnostic and prognostic studies: a tutorial. *Arquivos Brasileiros de Cardiologia*.

[35] Huang Z., Liu F. (2014). Diagnostic value of serum carbohydrate antigen 19-9 in pancreatic cancer: a meta-analysis. *Tumor Biology*.

[36] Walter S. D. (2002). Properties of the summary receiver operating characteristic (SROC) curve for diagnostic test data. *Statistics in Medicine*.

[37] Coory M. D. (2009). Comment on: heterogeneity in meta-analysis should be expected and appropriately quantified. *International Journal of Epidemiology*.

